# Predictors of health-related quality of life in a large cohort of adult patients living with sickle cell disease in France: the DREPAtient study

**DOI:** 10.3389/fpubh.2024.1374805

**Published:** 2024-05-20

**Authors:** Issifou Yaya, Adrien Pourageaud, Benjamin Derbez, Marie-Hélène Odièvre, Damien Oudin Doglioni, Marieke Podevin, Gaëlle Thomas, Lisa Yombo-Kokule, Christian Godart, Maryannick Lepetit, Tania Cassubie-Mercier, Frederic Galacteros, Olivier Chassany, DREPAtient study group, Marieke Podevin, Gaëlle Thomas, Olivier Chassany, Issifou Yaya, Lisa Yombo-Kokule, Frédéric Galactéros, Damien Oudin Doglioni, Odièvre-Montanié Marie-Hélène, Benjamin Derbez, Christian Godart, Maryannick Lepetit, Tania Cassubie-Mercier, Tania Cassubie-Mercier, Sonia Pavan, Patricia Aguilar-Martinez, Jean-Benoït Arlet, Giovanna Cannas, Abdourahim Chamouine, Maryse Etienne-Julan, Corinne Guitton, Sylvain Le Jeune, Gylna Loko, Corinne Pondarre

**Affiliations:** (ARGO Santé, Orléans, France); (URC-ECO, Hôpital Hotel-Dieu, AP-HP, Paris, France); (Unité des Maladies Génétiques du Globule rouge—Centre de référence maladie rare, Hôpital Henri Mondor, AP-HP, Créteil, France); (Centre de la drépanocytose, Hôpital Armand Trousseau, AP-HP, Paris, France); (LABERS/Dpt SHS, UFR Médecine, Brest, France); (Fédération nationale des associations de Malades Drépanocytaires et Thalassémiques (FMDT SOS GLOBI), Paris, France); (SOS GLOBI Rhône-Alpes, Le Péage-de-Roussillon, France); (Filière de santé MCGRE Hôpital Henri Mondor, AP-HP, Créteil, France); (Centre de référence syndromes drépanocytaires majeurs, thalassémies et autres maladies rares du globule rouge et de l’érythropoïèse Hôpital Saint Eloi, CHU Montpellier, Montpellier, France); (Centre de référence syndromes drépanocytaires majeurs, thalassémies et autres maladies rares du globule rouge et de l’érythropoïèse, Hôpital George Pompidou, AP-HP, Paris, France); (Centre de référence syndromes drépanocytaires majeurs, thalassémies et autres maladies rares du globule rouge et de l’érythropoïèse, Hôpital Edouard Herriot, Lyon, France); (Centre de référence syndromes drépanocytaires majeurs, thalassémies et autres maladies rares du globule rouge et de l’érythropoïèse, CHM, Mamoudzou Mayotte); (Centre de référence syndromes drépanocytaires majeurs, thalassémies et autres maladies rares du globule rouge et de l’érythropoïèse, Antilles-Guyane CHU de Pointe-à-Pitre/Abymes, Pointe-à-Pitre Guadeloupe); (Centre de référence syndromes drépanocytaires majeurs, thalassémies et autres maladies rares du globule rouge et de l’érythropoïèse, Hôpital Bicêtre, AP-HP, Le Kremlin-Bicêtre, France); (Centre de référence syndromes drépanocytaires majeurs, thalassémies et autres maladies rares du globule rouge et de l’érythropoïèse, Hôpital Avicenne, AP-HP, Bobigny, France); (Centre de référence syndromes drépanocytaires majeurs, thalassémies et autres maladies rares du globule rouge et de l’érythropoïèse Antilles-Guyane, CHU de Martinique, Le Lamentin, Martinique); (Centre de référence syndromes drépanocytaires majeurs, thalassémies et autres maladies rares du globule rouge et de l’érythropoïèse, Hôpital Créteil, AP-HP, Créteil, France); ^1^Patient-Reported Outcomes Research (PROQOL), Unité de Recherche Clinique en Economie de la Santé (URC-ECO), Hôpital Hôtel-Dieu, AP-HP, Paris, France; ^2^ECEVE, UMR-S 1123, Université Paris Cité, Inserm, Paris, France; ^3^CRESPPA-CSU, UMR 7217, Université Paris 8, Paris, France; ^4^Department of General Pediatrics, Sickle Cell Referal Center, Trousseau Hospital, AP-HP, Sorbonne Université, Paris, France; ^5^INSERM U1134, Integrated Red Globule Biology, Paris, France; ^6^Laboratoire Interuniversitaire de Psychologie/Personnalité, Cognition, Changement Social (LIP/PC2S), Université Grenoble Alpes, Grenoble, France; ^7^ARGO Santé, Orléans, France; ^8^Fédération nationale des associations de Malades Drépanocytaires et Thalassémiques SOS GLOBI (FMDT SOS GLOBI), Paris, France; ^9^Sickle Cell Referral Center, Internal Medicine Unit, Henri Mondor Hospital, AP-HP, U-PEC; INSERM-U955, Institut Mondor, Université Paris-Est Créteil, Team 2 Transfusion et Maladies du Globule Rouge, Créteil, France

**Keywords:** health-related quality of life, sickle cell disease, SF-36, France, DREPAtient study

## Abstract

**Background:**

Sickle cell disease (SCD) is an inherited autosomal recessive disorder exhibiting a range of symptoms and acute and/or chronic complications that affect the quality of life. This study aimed to assess health-related quality of life (HRQoL) and to identify the associated factors in adult patients with SCD in France.

**Methods:**

DREPAtient is a cross-sectional, multicenter study conducted from June 2020 to April 2021 in France and in certain French overseas territories where SCD is highly prevalent. Sociodemographic and clinical data were collected online. HRQoL was assessed by the French version of the 36-Item Short Form Survey (SF-36) questionnaire. HRQoL determinants were identified using multivariable linear regression analysis.

**Results:**

In total, 570 participants were included, mostly women (68.9%), with a mean age of 33.3 (±10.7) years. The highest mean score HRQoL was found in the Physical functioning domain (67.5 ± 21.8) and the lowest mean score in the General Health perception domain (37.7 ± 20.3). The mean score of the physical composite (PCS) and mental composite (MCS) of SF-36 summary scores was 40.6 ± 8.9 and 45.3 ± 9.8, respectively. Participants receiving oxygen therapy (*β* = −3.20 [95%CI: −5.56; −0.85]), those with a history of femoral osteonecrosis (−3.09 [−4.64; −1.53]), those hospitalized for vaso-occlusive crisis (VOC) or acute chest syndrome (ACS) (−2.58 [−3.93; −1.22]), those with chronic complications (−2.33 [−4.04; −0.62]), female participants (−2.17 [−3.65; −0.69]), those with psychological follow-up (−2.13 [−3.59; −0.67]), older participants (−1.69 [−3.28; −0.09]), and those receiving painkillers (−1.61 [−3.16; −0.06]) reported worse PCS score. By contrast, those who had completed secondary or high school (4.36 [2.41; 6.31]) and those with stable financial situation (2.85 [0.94, 4.76]) reported better PCS scores. Worse MCS scores were reported among participants with psychological follow-up (−2.54 [−4.28; −0.80]) and those hospitalized for VOC/ACS in the last 12 months (−2.38 [−3.99; −0.77]), while those who had relatives’ support (5.27 [1.92; 8.62]) and those with stable financial situation (4.95 [2.65; 7.26]) reported better MCS scores.

**Conclusion:**

Adults with major SCD reported poor physical and mental HRQoL scores. Hospitalization for VOC/ACS, chronic complications, use of painkillers, perceived financial situation, and support from relatives are important predictors of HRQoL in SCD patients. Interventions to improve HRQoL outcomes SCD should be considered.

## Background

Sickle cell disease (SCD) is one of the most common genetic blood disorders worldwide that affects the shape of red blood cells (1). It affects millions of people worldwide and is highly prevalent in populations in sub-Saharan Africa, South Asia, the Middle East, and the Mediterranean region. A recent comprehensive global assessment of prevalence showed that the number of people living with SCD in 204 countries and territories from 2000 (5.46 million) to 2021 (7.74 million) had globally increased by more than 40% (2).

SCD poses a great challenge to the health of the world’s population as it has a large impact on morbidity and mortality in children and adults. People with SCD may experience a wide range of symptoms, including pain, fatigue, vascular and extensive organ damage, and anemia (3, 4). SCD is also characterized by complications such as vaso-occlusive crisis (VOC) episodes, invasive infections, acute chest syndrome (ACS), strokes, and chronic pulmonary hypertension that could lead to death mostly in children under 5 years, adolescents, and pregnant women (5–7).

SCD has major public health socioeconomic implications for affected individuals, families, and communities. Indeed, chronic sickle cell crises may strongly impact the patient’s life conditions, particularly regarding education, work, psychosocial development, and health-related quality of life (HRQoL) in terms of physical and emotional wellbeing (8, 9). The HRQoL of a patient remains a determinant of health status related to chronic diseases, including SCD. Chronic pain is one of the most common symptoms experienced by people with SCD, which may lead to decreased physical activity, social isolation, and difficulty maintaining employment or pursuing education.

HRQoL in people with SCD is affected by multiple complex factors, including disease conditions as follows: stigma, discrimination, and lack of available treatment; family life and work problems; and social isolation. Prior studies (10, 11) among individuals with SCD have found that the group reported poorer HRQoL compared with the general population. In fact, in a literature review, SCD was found to be associated with poor HRQoL, impaired psychosocial functioning, and altered internal and interpersonal relationships (11). HRQOL scores in individuals living with SCD varied significantly across demographic and personal characteristics (age, gender, education, marital status, perceived stigma, health literacy, spirituality, and living situation) (4, 8, 12), but also SCD type or knowledge of SCD (13).

In France, SCD is considered as the most frequent genetic disease, and over 500 newborns are born with SCD each year, with almost 32,400 people living with SCD (14). Under the French Public Health Law of 2004, it was acknowledged as a national health priority. The HRQoL for people with SCD in France may vary depending on a range of factors, including the severity of their symptoms and how well their disease is managed, access to healthcare, availability of treatment options, and social support (14, 15).

Despite the challenges that many individuals may face with SCD, little is known about the HRQoL in that population. This study aimed to assess the HRQoL among adult individuals with SCD and identify its associated factors.

## Methods

### Study design and population

The DREPAtient was a large-scale survey designed to assess the HRQoL of patients with SCD, which included adults and children (and their parents). This study was a cross-sectional, multi-center, and nationwide study conducted in mainland France, as well as in certain overseas territories: Guadeloupe, Martinique, Réunion, Mayotte, and French Guiana. The investigating centers were Centres de Référence (CRMR) for major sickle cell syndromes, thalassemia, and other rare diseases of the red blood cell and erythropoiesis. The study information was disseminated mainly via the Internet, via the social networks of the organizations promoting the survey, and by sending communication kits (posters and flyers) to the reference centers.

The inclusion criteria for participation in the study were as follows: (1) adults with major SCD, or (2) having child(ren) with major SCD, (3) proficient in written and spoken French, and (4) consent to participate in the study.

### Data collection

Between June 2020 and April 2021, the data collection was carried out via the Modalisa platform, using a tested questionnaire developed in collaboration with a scientific committee comprising SCD experts, patients, and professionals specialized in survey methodologies. The questionnaire was completed online by the participants.

Socio-demographic data, medical history, including therapeutic data, information on complications (acute or chronic), and medical or surgical interventions (stay in intensive care, surgery, etc.) were collected as well as data on schooling (for children), professional life, and material and economic conditions. HRQoL was self-reported by patients using the generic 36-Item Short Form Survey (MOS SF-36, V1) (16).

### Variables definition

#### Outcome variable

The SF-36 questionnaire is a generic, multi-item self-assessment of HRQoL, consisting of 36 questions organized into 8 domains. The eight domains include physical functioning problems due to health issues (PF, 10 items), limitations caused by physical health disorders in role activities (RP, 4 items), bodily pain (BP, 2 items), perception of general wellbeing and health (GH, 5 items), vitality in fatigue and energy (VT, 4 items), limitations in social activities due to emotional and physical problems (SF, 2 items), general mental health (MH, 5 items), and limitations caused by emotions in role activities (RE, 3 items). The score of the eight domains of SF-36 ranged from 0 to 100 (best HRQoL). These eight domains were aggregated into two main scores, i.e., the Physical Component Summary score (PCS) and Mental Component Summary score (MCS) (17). All domains contributed different weights to the scoring of both PCS and MCS dimensions using special algorithms developed by Ware and colleagues (18). The two summary scores are normalized to an average of 50. In addition, the SF-36 questionnaire is not a sickle cell-specific related QoL assessment tool.

#### Explanatory variables

The explanatory variables concerned:

- Socio-demographic characteristics: sex (female/male), age (≤35/>35 years), born in France (yes/no), housing (living on own/living with parents), having a child (yes/no), having a sick child (yes/no), living in couple (yes/no), secondary or high school completed (yes/no), professionally active (yes/no), self-perceived financial situation (stable/moderate/unstable), and support received the relatives (yes/no),
- Clinical and therapeutic variables: hospitalization for VOC or ACS in the last 12 months (yes/no), admission in intensive care unit (ICU) in the last 12 months (yes/no), surgery, prosthesis or bone marrow transplant in the last 12 months (yes/no), blood transfusion in the last 12 months (yes/no), followed-up for other disease (yes/no), SCD-related treatment [hydroxycarbamide, oxygen therapy, and pain medications (yes/no)], psychological follow-up (yes/no), and acute or chronic complications. Acute complications referred to VOC, ACS, cerebrovascular accident, infectious complications (cholecystitis, pyelonephritis, osteomyelitis, and osteoarthritis), femoral osteonecrosis, priapism, auditory or visual complications, hepatitis, splenic sequestration, and acute anemia. However, chronic complications refer to pulmonary hypertension, cerebral vasculopathy, heart failure, renal insufficiency, retinopathy, skin ulcer, and severe anemia.

### Statistical data analysis

In the present study, only data from adults with major SCD were used.

A descriptive analysis of the characteristics of adults with SCD was performed. The socio-demographic, clinical, and therapeutic variables were calculated and presented as frequency and percentages for categorical variables as mean and standard deviation for continuous variables. In practice, the means of the scores of the eight domains and the two main dimensions (PCS and MCS) of the participants’ HRQoL were calculated. We used Student’s *t*-test or analysis of variance (ANOVA) to assess differences in PCS and MCS mean scores between subgroups of the respondents.

To investigate the determinants for HRQoL in the DREPatient study, we performed univariable linear regression models to test the association between participants’ characteristics and PCS or MCS. Significant variables with *p* < 0.25 were included in the multivariable linear regression models. After adjustment for age and sex, associations were considered statistically significant if the *p*-value was <0.05. Statistical analyses were carried out using R, version 4.2.2.

### Ethical considerations

Ethical approval was obtained from an independent ethics committee (Comité de Protection des Personnes, CPP Ouest V, approval number 20/030-3QE, May 2020). An informed consent was sought from the participants prior to answering the online questionnaire.

## Results

### Descriptive socio-demographic and clinical characteristics of the participants

**Figure 1:**
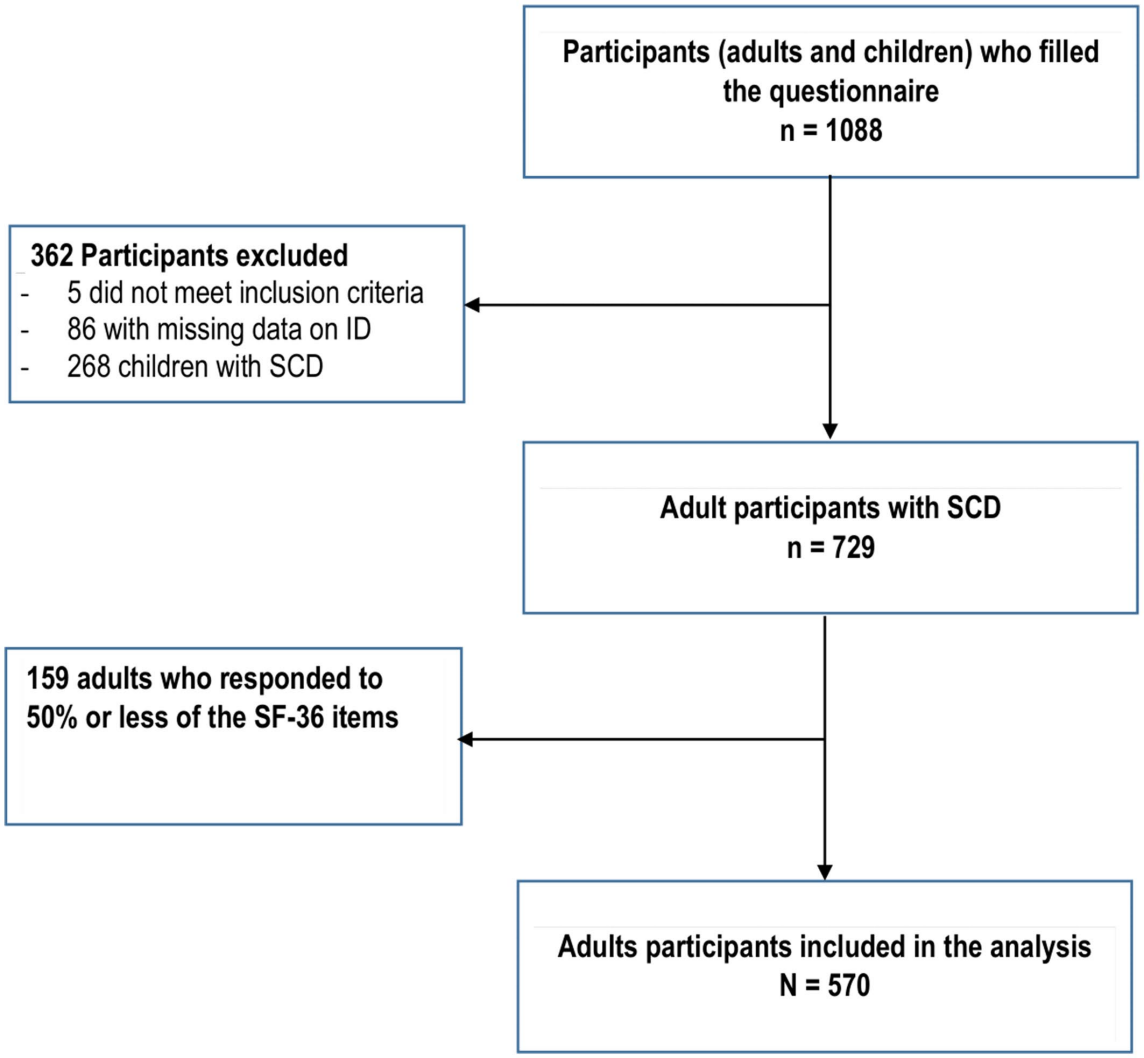
Flowchart.

Of the 1088 adults and children (and their parents) recruited for this study, 570 adults with major SCD were included in the analysis (Figure 1). Table 1 shows the socio-demographic and clinical data of the participants. Three-fifths (62.6%) of the participants were 35 years old or less, 384 (68.9%) of them were women and 53.9% were born in France. One-third of the participants (32.7%) were living with their parents or at least one family member, and 35.5% of the participants were living in couple. Most of the participants (85.2%) completed secondary or high school, 53.0% were professionally active, and 38.6% perceived themselves in a stable financial situation. In total, 216 participants (38.0%) reported having child(ren), and among them, 37 (17.6%) had child(ren) with SCD. Most of the participants (93.2%) reported having support (financial, material, moral, etc.) from relatives (parents, friends, etc.).

**Table 1:** Participants’ characteristics (*N* = 570).

Participants’ characteristics (*N* = 570)	*n* (%) mean (±SD)
**Sociodemographic variables**	
Sex (*n* = 557) Male Female	173 (31.1) 384 (68.9)
Age, years (*n* = 561) ≤35 >35	33.3 (±10.7) 351 (62.6) 210 (37.4)
Born in France (*n* = 566)	305 (53.9)
Housing (*n* = 566) By own means With parents	381 (67.3) 185 (32.7)
Living in couple (*n* = 564)	200 (35.5)
Having child(ren) (*n* = 568)	216 (38.0)
Child(ren) with SCD (*n* = 210)	37 (17.6)
Secondary or high school completed (*n* = 567)	484 (85.2)
Professional active (*n* = 547)	290 (53.0)
Self-perceived financial situation (*n* = 383) Unstable Moderate Stable	134 (35.0) 101 (26.4) 148 (38.6)
Relatives’ support (*n* = 570)	531 (93.2)
**Clinical and therapeutic variables**	
Hospitalization for VOC or ACS * (*n* = 566)	276 (48.8)
Admission in intensive care unit* (*n* = 561)	114 (20.3)
Surgery, prosthesis, or bone marrow transplant (*n* = 559)	216 (38.6)
Blood transfusion* (*n* = 565)	201 (35.6)
SCD-related treatment** (*n* = 551)	431 (78.2)
Follow-up for other disease (*n* = 567)	112 (19.8)
Psychological follow-up (*n* = 551)	179 (32.5)
Acute complications*** (*n* = 561)	542 (96.6)
Vaso-occlusive crisis (*n* = 543)	478 (88.0)
Acute chest syndrome (*n* = 501)	347 (69.3)
Stroke (*n* = 472)	44 (9.3)
Infectious complications (*n* = 479)	266 (55.5)
History of femoral osteonecrosis (*n* = 496)	149 (30.0)
Priapism (*n* = 480)	59 (12.3)
Auditory or visual complications (*n* = 509)	164 (32.2)
History of splenic sequestration (*n* = 485)	70 (14.4)
Acute anemia (*n* = 465)	313 (67.3)
Chronic complications**** (*n* = 478)	363 (75.9)
Pulmonary arterial hypertension (*n* = 458)	55 (12.0)
Cerebral vasculopathy (*n* = 447)	45 (10.1)
Heart failure (*n* = 495)	38 (7.7)
Renal failure (*n* = 482)	34 (7.1)
Retinopathy (*n* = 475)	89 (18.7)	Participants’ characteristics (*N* = 570)	*n* (%) mean (±SD)
Skin ulcer (*n* = 505)	77 (15.2)
Severe anemia (*n* = 468)	251 (53.6)

VOC, vaso-occlusive crisis; ACS, acute chest syndrome. * in the 12 last months. ** SCD-related treatment included hydroxycarbamide, oxygen therapy, or painkillers. *** Acute complications: Vaso-occlusive crisis (VOC), acute chest syndrome (ACS), cerebrovascular accident, infectious complications (cholecystitis, pyelonephritis, osteomyelitis, osteoarthritis), femoral osteonecrosis, priapism, auditory or visual complications, hepatitis, splenic sequestration, acute anemia. **** Chronic complications: Pulmonary hypertension, cerebral vasculopathy, heart failure, renal insufficiency, retinopathy, skin ulcer, severe anemia.

Almost half of the participants (48.8%) have already been hospitalized for VOC or ACS, one-fifth (20.3%) have been admitted to the intensive care unit, and one-third had a history of blood transfusion (35.6%) in the 12 last months. More than three-quarters of the participants (78.2%) received SCD-related treatment (including oxygen therapy, hydroxycarbamide, or painkillers-based treatments), and one-third (32.5%) had a psychological follow-up.

Regarding acute complications, most of the participants (96.6%) reported at least one complication, including VOC (88.0%), ACS (69.3%), acute anemia (67.3%), infectious complications (55.5%), auditory or visual complications (32.2%), femoral osteonecrosis (30.0%), and splenic sequestration (14.4%). Three-quarters of the participants (75.9%) reported at least one chronic complication, including severe anemia (53.6%), retinopathy (18.7%), skin ulcer (15.2%), pulmonary arterial hypertension (12.0%), and cerebral vasculopathy (10.1%) (Table 1).

Regarding characteristics of the participants, significant differences were observed between male and female participants, including having completed secondary or high school [79.2% for men and 88.2% for women (*p* = 0.005)], having support [89.6% for men and 94.8% for women (*p* = 0.024)], receiving psychological follow-up [25.0% for men and 36.5% for women (*p* = 0.009)], and history of splenic sequestration [20.2% for men and 11.9% for women (*p* = 0.013)].

### Description of the SF-36 domains scores

Table 2 shows the mean scores and standard deviation of 570 participants with SCD in the eight domains of HRQoL. In this study, the PF domain had the highest mean score (mean score: 67.5 ± 21.8), whereas the GH domain had the lowest mean score (37.7 ± 20.3). The Physical and Mental Component Summary scores were 40.6 ± 8.9 (PCS) and 45.8 ± 9.8 (MCS), respectively.

**Table 2:** Stratification of the SF-36 domains and component summary scores.

SF-36 domains	Mean ± SD	Min–max
Physical Functioning (PF), 10 items	67.5 ± 21.8	0–100
General Health (GH), 5 items	37.7 ± 20.3	0–100
Mental Health (MH), 5 items	67.3 ± 16.3	24–100
Role limitations due to Physical problems (RP), 4 items	49.9 ± 39.2	0–100
Vitality (VT), 4 items	53.7 ± 15.2	20–100
Role limitations due to Emotional problems (RE), 3 items	56.3 ± 42.6	0–100
Bodily Pain (BP), 2 items	67.4 ± 24.0	20–100
Social Functioning (SF), 2 items	66.1 ± 21.8	12.5–100
Physical component summary score (PCS)	40.6 ± 8.9	16.1–61.4
Mental component summary score (MCS)	45.3 ± 9.8	15.8–67.5

SF-36 domain range scores: 0–100 (best HRQoL). Component Summary scores were normalized to an average of 50.

The relationship between HRQoL domains and, socio-demographic and clinical characteristics of the participants are shown in Supplementary Table S1. The mean PCS and MCS scores were lower among participants with unstable or moderate financial situations, those hospitalized for VOC or ACS, those admitted to ICU, those who received painkillers, those in psychological follow-up, and those who reported acute or chronic complications. In addition, those being professionally active and those with relatives’ support reported higher mean scores for the PCS and MCS (Supplementary Table S1).

### Factors associated with HRQoL

#### Physical component summary score

In the univariable analysis (Table 3), sex, age, education level, self-perceived financial situation, professional activity, relatives’ support, hospitalization for VOC or ACS, admission in ICU, history of surgery, prosthesis or bone marrow transplant, history of blood transfusion, receiving SCD-related treatments, including oxygen therapy or use of painkillers, psychological follow-up, history of femoral osteonecrosis, and presence of or chronic complications were significantly associated with PCS score.

**Table 3:** Linear regression model of factors associated with health-related quality of life (*N* = 570).

	Linear regression for PCS		Linear regression for MCS	
	Univariable *β* [95% CI]	Multivariable *β* [95% CI]	Univariable *β* [95% CI]	Multivariable Ψ *β* [95% CI]
Sex, female	−2.22 [−3.86; −0.57]	−2.17 [−3.65; −0.69]	−0.73 [−2.55; 1.08]	
Age > 35 years	−3.75 [−5.29; −2.22]	−1.69 [−3.28; −0.09]	0.80 [−0.92; 2.52]	
Born in France	0.79 [−0.73; 2.32]		1.43 [−0.24; 3.10]	
Living in couple	−1.41 [−2.99; 0.17]		0.72 [−1.01; 2.45]	
Child(ren) with SCD	2.73 [−0.58; 6.04]		−0.04 [−3.75; 3.67]	
Secondary or high school completed	5.70 [3.63; 7.77]	4.36 [2.41; 6.31]	0.27 [−2.06; 2.60]	
Self-perceived financial situation Unstable Moderate Stable	0 2.27 [−0.01; 4.54] 4.99 [2.92; 7.06]	0 0.36 [−1.73; 2.44] 2.85 [0.94; 4.76]	0 1.84 [−0.66; 4.35] 6.46 [4.18; 8.73]	0 1.07 [−1.42; 3.56] 4.95 [2.65; 7.26]
Professionally active	2.24 [0.71; 3.77]		2.88 [1.20; 4.55]	
Relatives’ support	4.15 [1.07; 7.24]		6.95 [3.60; 10.30]	5.27 [1.92; 8.62]
Hospitalization for VOC or ACS*	−3.83 [−5.31; −2.34]	−2.58 [−3.93; −1.22]	−3.16 [−4.80; −1.52]	−2.38 [−3.99; −0.77]
Admission in intensive care unit*	−3.63 [−5.52; −1.74]		−2.23 [−4.32; −0.14]	
Surgery, prosthesis or bone marrow transplant	−2.91 [−4.45; −1.36]		−0.28 [−2.00; 1.43]	
History of blood transfusion*	−2.36 [−3.94; −0.79]		−0.80 [−2.53; 0.94]	
Follow-up for other disease	−1.91 [−3.80; −0.01]		−0.73 [−2.81; 1.36]	
SCD-related treatment**	−5.99 [−7.76; −4.22]		−1.44 [−3.44; 0.57]	
Hydroxycarbamide use	1.63 [−0.15; 3.40]		2.02 [0.01; 4.03]	
Oxygen therapy	−5.36 [−7.85; −2.87]	−3.20 [−5.56; −0.85]	−1.39 [−4.26; 1.48]	
Use of painkillers	−2.87 [−4.53; −1.20]	−1.61 [−3.16; −0.06]	−2.77 [−4.66; −0.88]	
Psychological follow-up	−3.86 [−5.46; −2.25]	−2.13 [−3.59; −0.67]	−3.30 [−5.07; −1.54]	−2.54 [−4.28; −0.80]
History of femoral osteonecrosis	−4.61 [−6.33; −2.89]	−3.09 [−4.64; −1.53]	−0.06 [−1.98; 1.86]	
History of splenic sequestration	−2.11 [−4.46; 0.25]		−0.24 [−2.83; 2.35]	
Chronic complications***	−4.43 [−6.30; −2.57]	−2.33 [−4.04; −0.62]	−2.17 [−4.25; −0.09]	

PCS = physical component summary; MCS = mental component summary; SCD = sickle cell disease; VOC = vaso-occlusive crisis; ACS = acute chest syndrome; Ψ: adjusted for sex and age. * In the 12 last months. ** SCD-related treatment included hydroxycarbamide, oxygen therapy, or painkillers. *** Chronic complications: Pulmonary hypertension, cerebral vasculopathy, heart failure, renal insufficiency, retinopathy, skin ulcer, severe anemia.

Multivariable analysis showed that participants receiving oxygen therapy (*β* = −3.20 [−5.56; −0.85]), those with a history of femoral osteonecrosis (*β* = −3.09 [−4.64; −1.53]), those hospitalized for VOC or ACS (*β* = −2.58 [−3.93; −1.22]), those with chronic complications (*β* = −2.33 [−4.04; −0.62]), female participants (*β* = −2.17 [95%CI, −3.65, −0.69]), those who reported psychological follow-up (*β* = −2.13 [−3.59; −0.67]), those aged more than 35 years (−1.69 [−3.28; −0.09]), and those who reported painkillers use (*β* = −1.61 [−3.16; −0.06]) reported worse score in the PCS dimension. By contrast, participants who have completed secondary or high school (4.36 [2.41, 6.31]), and those who perceived themselves in a stable financial situation (*β* = 2.85 [0.94, 4.76]) reported better scores in the PCS dimension (Table 3).

#### Mental component summary score

The univariable analysis highlighted that self-perceived financial situation, professional activity, relatives’ support, hospitalization for VOC or ACS, admission in ICU, receiving hydroxycarbamide or painkillers, psychological follow-up, and presence of chronic complications were significantly associated with MCS score (Table 3).

In the multivariable analysis, participants who reported psychological follow-up (*β* = −2.54 [−4.28; −0.80]) and those hospitalized for a VOC or ACS in the last 12 months (*β* = −2.38 [−3.99; −0.77]) had worse scores in the MCS dimension. By contrast, those who had relatives’ support (*β* = 5.27 [1.92, 8.62]) and those who perceived themselves in a stable financial situation (*β* = 4.95 [2.65, 7.26]) reported better scores in the MCS dimension (Table 3).

## Discussion

The aim of this study was to evaluate HRQoL using the SF-36 in a large French sample of 570 adults with SCD. The results indicate poor HRQoL scores, more marked in the RP and GH domains. Globally, the PCS score (40.6 ± 8.9) was more affected than the MCS score (45.3 ± 9.8). Our finding is consistent with studies by Dampier et al. and McClish et al. in the USA (8, 9). However, lower scores were reported in some Middle east countries by Ahmadi et al. (19) and Ahmed et al. (20). The HRQoL scores in our study seemed to be lower than those reported in the French general population (50.2 ± 9.1 for the PCS score and 47.2 ± 9.7 for the MCS score) according the French Decennial Health Survey, but higher than that in individuals with other chronic conditions, including chronic kidney disease (QUAVI-REIN study) (21). Comparison of these data might be difficult even if identical tools had been used, as it was based in part on data collected in 2003 (21), which could raise temporal issues. Since the collection of these data, medical advances in the field of SCD management including medications, blood transfusions, and bone marrow transplants may have reduced the frequency and severity of complications and thus improved HRQoL.

This study highlighted several factors that need attention and a better management in order to improve HRQoL in adults with SCD.

First, female or older participants, those who have been hospitalized for VOC or ACS in the last 12 months, those with psychological follow-up, and those with chronic complications reported worse PCS or MCS scores. Similar to the previous studies on HRQoL among patients with SCD, female participants in our study experienced poor HRQoL, in physical dimensions. A hallmark of SCD, pain crises are common and may be debilitating, which can significantly impact HRQoL (22). Perception and experience of pain can vary between individuals, and there is evidence to suggest that there are gender differences in how pain is perceived and reported. These differences can be influenced by various biological, psychological, and sociocultural factors (23–25). Female participants with SCD may experience pain during menstruation, which can exacerbate their overall pain burden (26, 27). Older participants with SCD may experience an increasing risk of SCD-related complications such as organ damage, stroke, and infections (28) that could severely affect their HRQoL. Managing these complications through regular medical check-ups and preventive measures is essential for maintaining a higher quality of life.

Chronic pain in adults with SCD can have a physical impact, leading to fatigue, reduced mobility, and difficulty performing daily activities. Thus, in our study, adults with SCD who were receiving pain medications or oxygen therapy reported worse scores for PCS. Receiving pain medications constitutes a proxy for the presence of chronic pain reported by patients, regardless of its intensity. Several studies on HRQoL among patients living with SCD showed similar findings (8, 29). A study by Dampier et al. found that pain impairs HRQoL more than any other SCD-related complications (8).

Second, SCD is known for causing severe, recurrent episodes of pain, known as vaso-occlusive crises (VOC). These could be excruciating and may require hospitalization and strong pain medications. This study demonstrated that in adults with SCD, hospitalization for VOC and/or ACS has a poor impact on overall HRQoL (PCS and MCS). These findings in our study are in line with previous studies highlighting that hospitalization for VOC had a negative impact on HRQoL in patients with SCD (30–33). Although medications and advanced medical technologies remain effective for the management of SCD (34), patients may have a traumatic experience of hospitalization, especially when it is recurrent. The burden of repeated hospitalizations can lead to psychosocial challenges, including anxiety, depression, and impaired HRQoL. We have little perspective and no information on the length of the hospital stay in our study; however, the impact of hospitalization on HRQoL may persist for up to a year, and due to the presence of sequelae and complications, especially chronic complications. As highlighted in our study, several studies found that individuals with SCD can experience a wide range of complications that may negatively impact their HRQoL (8, 9, 35, 36). The severity and frequency of these complications can vary from person to person with SCD (37). The complications of SCD include anemia, functional asplenia, strokes, and pain crises, which are caused by hemolysis and/or vascular occlusion (34). Those individuals need recurrent hospital stays with chronic treatment and frequent follow-up visits, including psychological follow-up, as shown in our findings. Psychological follow-up may be an essential component of the holistic care for individuals with SCD, especially considering the chronic nature of the disease and its impact on HRQoL. Thus, advances in medical management have contributed to an increase in life expectancy for many individuals with SCD. Our findings suggested that regular medical check-ups, preventive measures, and appropriate treatments in order to manage these complications could help to improve the HRQoL for individuals with SCD.

Third, similar to results from recent studies among patients with SCD (38, 39), higher education level was found to be associated with better scores in the PCS dimension in the present study. This association may be mediated by higher level of health literacy, good knowledge, and positive perception regarding SCD. In addition, higher educational attainment will help patients to improve their capacity for self-management of the SCD and the prevention of episodes of VOC and complications (40). Moreover, education level may often correlate with socioeconomic status and could be considered as a proxy for the level of health literacy, which could impact access to resources, healthcare, and overall wellbeing; as shown in our study, participants with stable financial situation were more likely to reported higher scores in the PCS and MCS dimensions. Efforts to promote education and health literacy, alleviate poverty, and provide equitable access to healthcare are essential components of public health initiatives aiming to reduce health inequalities.

Finally, support such as that offered by family members and friends is crucial for individuals with SCD to help them cope with the physical, emotional, and social challenges associated with the condition and improve the overall HRQoL (41). This support includes participation in social activities, maintaining a positive social network, and reducing feelings of isolation (42). Indeed, our findings revealed that participants who benefited from support from relatives reported better scores for MCS. That is line with several studies concluding that greater parental support and involvement is significantly associated to decreased depressive symptoms and better HRQoL in patients with SCD, particularly in children (43–45). Although we did not explore various types of social support in our study, findings may suggest the need of advance understanding of the role of relatives’ support in the lives of individuals with SCD. This indicates that support intervention is an important part of the management strategies of adults with SCD, including family or relatives’ support that leads to improving HRQoL.

Some limitations of this study should be mentioned. The sample did not include non-French-speaking participants or no internet users and may lack representativeness among patients with low health literacy. The observed statistical associations in this cross-sectional study may not necessarily imply causality. Data on depression, anxiety and psychological issues were not collected, which could have been useful to explore another aspect of participants’ wellbeing. SF-36, a generic questionnaire, may not be the most relevant questionnaire compared to SCD-specific questionnaires. However, SF-36 has been used in SCD studies and is able to capture the physical and psychological impact of the disease, especially as the impact of SCD is significant. Indeed, the low SF-36 scores and the factors associated, which are reported in this study, may be useful not only to patients with SCD but also to healthcare workers who take care of these patients.

## Conclusion

Findings from this study indicated that adults with SCD have poor physical and mental HRQoL scores. Women, older participants, and those who were hospitalized for VOC and/or ACS or other complications experienced worse scores for HRQoL. These findings suggest paying greater attention to HRQoL in adults with SCD, in terms of SCD management strategies. Future research should investigate and better understand the HRQoL evolution during multiple phases of the disease and its determinants.

## Data availability statement

The datasets presented in this article are not readily available because the data that support the findings of this study are available on request from OC. The data are not publicly available due to confidentiality restrictions. Requests to access the datasets should be directed to OC, olivier.chassany@aphp.fr.

## Ethics statement

The studies involving humans were approved by Comité de Protection des Personnes, CPP Ouest V, approval number 20/030-3QE, May 2020. The studies were conducted in accordance with the local legislation and institutional requirements. Written informed consent for participation in this study was provided by the participants’ legal guardians/next of kin.

## Author contributions

IY: Conceptualization, Data curation, Formal analysis, Methodology, Software, Writing – original draft. AP: Data curation, Formal analysis, Methodology, Software, Writing – review & editing. BD: Writing – review & editing. M-HO: Writing – review & editing. DO: Writing – review & editing. MP: Funding acquisition, Investigation, Methodology, Project administration, Writing – review & editing. GT: Funding acquisition, Investigation, Methodology, Project administration, Writing – review & editing. LY-K: Data curation, Formal analysis, Writing – review & editing. CG: Writing – review & editing. ML: Writing – review & editing. TC-M: Writing – review & editing. FG: Investigation, Writing – review & editing. OC: Conceptualization, Supervision, Writing – review & editing.

## Group member of the DREPAtient study group

Marieke Podevin and Gaëlle Thomas (ARGO Santé, Orléans, France), Olivier Chassany, Issifou Yaya, and Lisa Yombo-Kokule (URC-ECO, Hôpital Hotel-Dieu, AP-HP, Paris, France), Frédéric Galactéros and Damien Oudin Doglioni (Unité des Maladies Génétiques du Globule rouge—Centre de référence maladie rare, Hôpital Henri Mondor, AP-HP, Créteil, France), Odièvre-Montanié Marie-Hélène (Centre de la drépanocytose, Hôpital Armand Trousseau, AP-HP, Paris, France), Benjamin Derbez (LABERS/Dpt SHS, UFR Médecine, Brest, France), Christian Godart, Maryannick Lepetit, and Tania Cassubie-Mercier (Fédération nationale des associations de Malades Drépanocytaires et Thalassémiques (FMDT SOS GLOBI), Paris, France), Tania Cassubie-Mercier (SOS GLOBI Rhône-Alpes, Le Péage-de-Roussillon, France), Sonia Pavan (Filière de santé MCGRE Hôpital Henri Mondor, AP-HP, Créteil, France), Patricia Aguilar-Martinez (Centre de référence syndromes drépanocytaires majeurs, thalassémies et autres maladies rares du globule rouge et de l’érythropoïèse Hôpital Saint Eloi, CHU Montpellier, Montpellier, France), Jean-Benoït Arlet (Centre de référence syndromes drépanocytaires majeurs, thalassémies et autres maladies rares du globule rouge et de l’érythropoïèse, Hôpital George Pompidou, AP-HP, Paris, France), Giovanna Cannas (Centre de référence syndromes drépanocytaires majeurs, thalassémies et autres maladies rares du globule rouge et de l’érythropoïèse, Hôpital Edouard Herriot, Lyon, France), Abdourahim Chamouine (Centre de référence syndromes drépanocytaires majeurs, thalassémies et autres maladies rares du globule rouge et de l’érythropoïèse, CHM, Mamoudzou Mayotte), Maryse Etienne-Julan (Centre de référence syndromes drépanocytaires majeurs, thalassémies et autres maladies rares du globule rouge et de l’érythropoïèse, Antilles-Guyane CHU de Pointe-à-Pitre/Abymes, Pointe-à-Pitre Guadeloupe), Corinne Guitton (Centre de référence syndromes drépanocytaires majeurs, thalassémies et autres maladies rares du globule rouge et de l’érythropoïèse, Hôpital Bicêtre, AP-HP, Le Kremlin-Bicêtre, France), Sylvain Le Jeune (Centre de référence syndromes drépanocytaires majeurs, thalassémies et autres maladies rares du globule rouge et de l’érythropoïèse, Hôpital Avicenne, AP-HP, Bobigny, France), Gylna Loko (Centre de référence syndromes drépanocytaires majeurs, thalassémies et autres maladies rares du globule rouge et de l’érythropoïèse Antilles-Guyane, CHU de Martinique, Le Lamentin, Martinique), Corinne Pondarre (Centre de référence syndromes drépanocytaires majeurs, thalassémies et autres maladies rares du globule rouge et de l’érythropoïèse, Hôpital Créteil, AP-HP, Créteil, France).
