# Supplementary material for: Predictors of health-related quality of life in a large cohort of adult patients living with sickle cell disease in France: the DREPAtient study
Source: Front Public Health. 2024 May 20;12:1374805. doi: 10.3389/fpubh.2024.1374805 (PMC11144927; doi:10.3389/fpubh.2024.1374805)
Supplement: Supplementary file 1 [file Table_1.docx]

**Supplementary Table 1: Participants’ characteristics**

|  | **PCS** | | **MCS** | |
| --- | --- | --- | --- | --- |
|  | **Mean (±SD)** | **p-value^a^** | **Mean (±SD)** | **p-value^a^** |
| **Sociodemographic variables** | | | | |
| **Sex (n= 526)**  *Male*  *Female* | 42.2 (8.3)  40.0 (9.0) | **0.008** | 45.8 (9.9)  45.0 (9.7) | 0.427 |
| **Age, years (n= 538)**  *< 35*  *≥35* | 42.1 (8.7)  38.4 (8.8) | **< 0.001** | 44.8 (10.3)  46.0 (8.9) | 0.190 |
| **Born in France (n= 534)**  *No*  *Yes* | 40.2 (8.8)  41.0 (9.0) | 0.308 | 44.5 (9.9)  45.9 (9.6) | 0.093 |
| **Housing (n= 536)**  *By own means*  *With parents* | 39.6 (9.2)  43.0 (8.0) | **< 0.001** | 45.9 (9.9)  45.0 (9.7) | 0.357 |
| **Living in couple (n= 533)**  *No*  *Yes* | 41.2 (8.9)  39.8 (8.9) | 0.079 | 45.0 (9.9)  45.7 (9.6) | 0.416 |
| **Having child(ren) (n= 536)**  *No*  *Yes* | 42.1 (8.8)  38.4 (8.7) | **< 0.001** | 45.3 (10.1)  45.3 (9.2) | 0.984 |
| **Child(ren) with SCD (n= 200)**  *No*  *Yes* | 38.0 (8.6)  40.7 (8.5) | 0.099 | 45.3 (9.3)  45.3 (9.1) | 0.983 |
| **Secondary or high school completed (n= 535)**  *No*  *Yes* | 35.8 (8.8)  41.5 (8.7) | **< 0.001** | 45.0 (8.6)  45.3 (10.0) | 0.821 |
| **Professional active (n= 521)**  *No*  *Yes* | 39.4 (9.1)  41.6 (8.7) | **< 0.001** | 43.7 (9.8)  46.6 (9.3) | **0.001** |
| **Self-perceived financial situation (n=371)**  *Stable*  *Average*  *Unstable* | 42.0 (9.1)  39.3 (9.2)  37.0 (8.4) | **< 0.001^b^** | 48.4 (8.9)  43.8 (9.5)  41.9 (9.6) | **< 0.001^b^** |
| **Relatives’ support (n=538)**  *No*  *Yes* | 36.7 (9.0)  40.9 (8.9) | **0.009** | 38.8 (8.4)  45.7 (9.7) | **< 0.001** |
| **Clinical and therapeutic variables** | | | | |
| **Hospitalization for VOC or ACS * (n= 535)**  *No*  *Yes* | 42.5 (9.0)  38.7 (8.3) | **< 0.001** | 46.8 (9.5)  43.6 (9.8) | **< 0.001** |
| **Admission in intensive care unit* (n= 531)**  *No*  *Yes* | 41.3 (8.8)  37.7 (8.7) | **< 0.001** | 45.8 (9.7)  43.5 (10.0) | **0.037** |
| **Surgery, prosthesis or bone marrow transplant (n= 529)**  *No*  *Yes* | 41.8 (8.6)  38.9 (9.1) | **< 0.001** | 45.4 (9.8)  45.1 (9.8) | 0.746 |
| **Blood transfusion* (n=533)**  *No*  *Yes* | 41.5 (9.1)  39.1 (8.5) | **0.003** | 45.5 (10.2)  44.8 (9.0) | 0.368 |
| **Follow-up for other disease (n=535)**  *No*  *Yes* | 41.0 (9.1)  39.1 (8.3) | **0.049** | 45.5 (9.8)  44.7 (9.5) | 0.492 |
| **SCD-related treatment** (n=520)**  *No*  *Yes* | 45.2 (8.2)  39.2 (8.7) | **< 0.001** | 46.2 (9.4)  44.8 (9.9) | 0.163 |
| **Hydroxycarbamide use (n= 405)**  *No*  *Yes* | 38.2 (8.6)  39.8 (8.7) | 0.076 | 43.5 (9.7)  45.5 (10.0) | 0.052 |
| **Oxygen therapy (n= 405)**  *No*  *Yes* | 39.9 (8.5)  34.6 (8.9) | **<0.001** | 45.0 (10.0)  43.6 (8.9) | 0.350 |
| **Painkillers use (n= 405)**  *No*  *Yes* | 40.7 (8.9)  37.9 (8.3) | **<0.001** | 46.2 (9.3)  43.5 (10.3) | **0.005** |
| **Psychological follow-up (n= 521)**  *No*  *Yes* | 41.9 (8.9)  38.1 (8.5) | **< 0.001** | 46.5 (9.7)  43.2 (9.7) | **< 0.001** |
| **Complication variables** | | | | |
| **Acute complications*** (n=530)**  *No*  *Yes* | 48.5 (8.6)  40.3 (8.8) | **0.001** | 48.8 (7.6)  45.2 (9.8) | 0.121 |
| **Vaso-occlusive crisis (n=515)**  *No*  *Yes* | 44.3 (8.8)  40.0 (8.9) | **< 0.001** | 48.6 (7.9)  44.9 (9.9) | **0.004** |
| **Acute chest syndrome (n=477)**  *No*  *Yes* | 43.5 (8.3)  38.8 (8.9) | **< 0.001** | 46.6 (8.9)  45.1 (10.1) | 0.126 |
| **Stroke (n=450)**  *No*  *Yes* | 41.4 (8.9)  37.8 (8.3) | **0.014** | 45.6 (9.7)  46.6 (9.9) | 0.538 |
| **Infectious complications (n=457)**  *No*  *Yes* | 42.9 (9.4)  38.7 (8.2) | **< 0.001** | 45.6 (10.0)  45.2 (9.53) | 0.607 |
| **History of femoral osteonecrosis (n=471)**  *No*  *Yes* | 41.9 (8.8)  37.3 (8.3) | **< 0.001** | 45.7 (10.0)  45.6 (9.4) | 0.952 |
| **Priapism (n=460)**  *No*  *Yes* | 40.9 (8.8)  41.3 (9.2) | 0.773 | 45.6 (9.7)  44.4 (9.3) | 0.379 |
| **Auditory or visual complications (n=488)**  *No*  *Yes* | 41.7 (9.0)  38.6 (8.8) | **< 0.001** | 46.2 (9.8)  44.3 (9.5) | **0.038** |
| **History of splenic sequestration (n=464)**  *No*  *Yes* | 40.9 (8.8)  38.8 (9.0) | 0.078 | 45.4 (9.7)  45.2 (10.4) | 0.854 |
| **Acute anemia (n= 441)**  *No*  *Yes* | 43.5 (9.2)  38.9 (8.5) | **< 0.001** | 47.7 (9.3)  44.0 (9.7) | **< 0.001** |
| **Chronic complications**** (n=457)**  *No*  *Yes* | 43.6 (9.0)  39.2 (8.7) | **< 0.001** | 47.2 (9.0)  45.0 (9.9) | **0.040** |
| **Pulmonary arterial hypertension (n=439)**  *No*  *Yes* | 41.3 (9.0)  38.9 (8.8) | 0.067 | 46.2 (9.6)  44.5 (9.8) | 0.216 |
| **Cerebral vasculopathy (n=432)**  *No*  *Yes* | 41.3 (8.9)  37.7 (7.8) | **0.012** | 46.2 (9.6)  43.8 (10.2) | 0.113 |
| **Heart failure (n=475)**  *No*  *Yes* | 41.0 (8.9)  36.0 (8.8) | **0.001** | 45.7 (9.7)  44.7 (8.9) | 0.535 |
| **Renal failure (n=462)**  *No*  *Yes* | 41.0 (8.9)  37.6 (8.9) | **0.036** | 45.7 (9.8)  44.3 (8.1) | 0.409 |
| **Retinopathy (n=455)**  *No*  *Yes* | 41.2 (9.0)  39.0 (8.5) | **0.042** | 45.6 (9.8)  44.5 (10.0) | 0.331 |
| **Skin ulcer (n=483)**  *No*  *Yes* | 41.0 (8.7)  38.4 (9.5) | **0.020** | 45.4 (9.7)  46.6 (9.7) | 0.323 |
| **Severe anemia, Hb < 7 (n=446)**  *No*  *Yes* | 43.1 (8.7)  38.4 (8.6) | **< 0.001** | 47.0 (9.4)  45.5 (9.7) | **0.005** |

a: student t-test; b: ANOVA

VOC = vaso-occlusive crisis ; ACS = acute chest syndrome

* In the 12 last months

** SCD-related treatment included Oxygen therapy, hydroxycarbamide, painkillers

*** Acute complications: Vaso-occlusive crisis (VOC), acute chest syndrome (ACS), cerebrovascular accident, infectious complications (cholecystitis, pyelonephritis, osteomyelitis, osteoarthritis), femoral osteonecrosis, priapism, auditory or visual complications, hepatitis, splenic sequestration, acute anemia)

**** Chronic complications: Pulmonary hypertension, cerebral vasculopathy, heart failure, renal insufficiency, retinopathy, skin ulcer, severe anemia
